# Possibility for Replicating Mechanoscopic Surface Marks in the Hybrid Vacuum-Pressure Casting Process

**DOI:** 10.3390/polym13060874

**Published:** 2021-03-12

**Authors:** Mariusz Frankiewicz, Karol Kobiela, Tomasz Kurzynowski

**Affiliations:** Centre for Advanced Manufacturing Technologies (CAMT-FPC), Faculty of Mechanical Engineering, Wroclaw University of Science and Technology, Lukasiewicza 5, 50-371 Wrocław, Poland; karol.kobiela@pwr.edu.pl (K.K.); tomasz.kurzynowski@pwr.edu.pl (T.K.)

**Keywords:** polymer solution casting, room temperature vulcanization (RTV), surface marks replication

## Abstract

Vacuum-pressure casting technology allows small batches of components to be manufactured from polymer materials, mainly from thermosetting plastics such as polyurethane and epoxy resins. Apart from being very simple, the process is also advantageous in that it offers a very accurately reproduced geometrical structure of the surfaces of master patterns used in mold manufacturing. This article presents the results of analyses performed for the process of replicating mechanoscopic marks with the use of three vacuum casting variants, including a hybrid vacuum-pressure casting process developed in particular for the replication purposes. The main research objective was to analyze and evaluate the influence of the parameters of the individual process variants on the quality of the obtained cast parts and on the replication accuracy without introducing additional artifacts on their surfaces. The article discusses the individual stages of the process and provides an analysis of their parameters. The replicas were evaluated for their porosity and reproduction quality with the use of CT methods and comparative photographs obtained from a light microscope.

## 1. Introduction

### State of the Art—Vacuum Casting (VC)

For more than two decades, the Vacuum Casting (VC) process has been widely used, in particular for small-batch and unit production [1]. Low manufacturing costs and short manufacturing time, as well as the tool-forming technique allow the process to be classified in the group of indirect rapid tooling methods, which shorten the time-to-market in the development of new products [2,3,4]. It consists of vacuum casting of thermosetting epoxy or polyurethane resins, as well as wax, in elastomer (silicone) molds. The tools most frequently used in the VC process include silicone rubber molds manufactured in Room Temperature Vulcanization (RTV) by the encapsulation of the master pattern [5,6]. The principle behind the RTV method is to reproduce the geometry of the master pattern (most often additively manufactured) by encapsulating it with two-component silicone rubbers, of an additive or polycondensation type. The RTV process is typically performed in atmospheric pressure. However, in order to ensure a highly accurate reproduction of the pattern surface, a vacuum degassing stage is introduced directly after the pattern is encapsulated in the silicone rubber. It allows the removal of gas bubbles formed during the mixing of silicone components due to the high viscosity of silicone rubber. Both the RTV and the VC may be thus classified as replication processes [7].

Research works demonstrate that the VC methods, owing to their very high reproduction accuracy, are used in micro-scale manufacturing. D.Y. Zhao et al. investigated the replication accuracy in complex surface microstructures using negative molds obtained in the vacuum casting process and demonstrated ability of transferring complex structures of micro-riblets to silicone film with replication errors below 5 µm [8]. Those authors also underlined that vacuum parameters played significant role in replication process and influencing the replication quality but dependency between numbers of pores on the surface on and vacuum parameters has no being discussed. Y. Tang et al. analyzed the geometric reproduction accuracy of micro-molds manufactured in the VC process and confirm low deviations of the dimensions below 10 µm and the roughness of the replicated surface below 0.5 nm [5]. M. Denoual et al. applied the VC method to obtain MEMS structures used in medical analyses and confirm potential of high accuracy of replications of submicron features [9]. L., S. Desmet et al. analyzed the accuracy of vacuum cast concave refractive microlens arrays and by high precision of the VC method achieved good optical quality of the manufactured objects [10].

The research results demonstrate that the VC technology has a considerable application potential in areas where the quality of reproduced external surfaces is of key importance [2,8,9,10,11,12,13,14,15,16]. Therefore, the authors decided to use this manufacturing method in their research on manufacturing accurate replicas of mechanoscopic forensic marks. The modification of the process by providing an additional post-processing stage which consists in pressure curing allowed a simplified manufacturing process while preserving high reproduction quality.

## 2. Aim of Research

All of the above-mentioned works stress the potential for obtaining high reproduction quality. The VC technology offers a highly accurate replication of the geometrical structures of master pattern surfaces, and therefore may be used in such applications as preparing copies of mechanoscopic marks left by firearms on cartridge cases and bullets. The current standards in the forensic examinations and identification of firearms used in crimes still rely on microscope-based methods of comparing marks on the surfaces of actual objects (evidence). The implementation of VC in preparing replicas of this type of evidence allows accurate copies of e.g., cases or bullets to be shared and used as a basis for sets of evidence material collected in a number of locations. This improvement provides an opportunity to identify and profile firearms used in crimes faster and within a wider range by examining such characteristic features as marks left by the magazine lips, receiver, the firing pin, the ejector, or the extractor. Its simplicity, cost-effectiveness, and speed are among the advantages of practically applying the VC replication method in the discussed field.

## 3. Materials and Design

**Figure 1:**
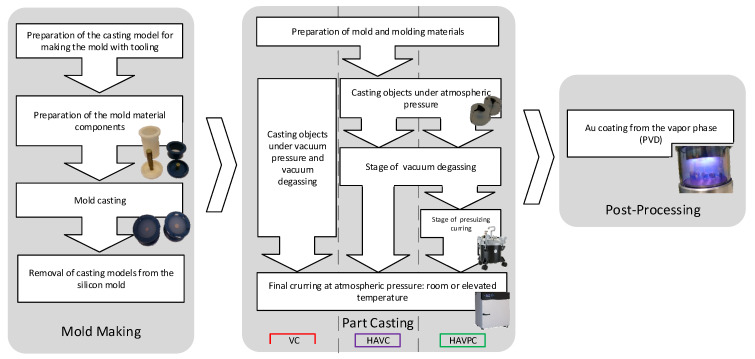
Schematic diagram of the technological process for replicating forensic marks.

The proposed process for the production of replicas is performed in three stages (Mold Making, Part Casting, Post-Processing), as shown in Figure 1. The research was also divided into three parts, corresponding to the successive stages of the replication process. The first part consisted in the development of a method for manufacturing a forming tool which enables the replication of desired mechanoscopic characteristics. The second part involved the development and evaluation of the replica production process, including the identification and verification of a set of process parameters. The final, third part was the evaluation of the additional post-processing treatment, which included the application of a Physical Vapor Deposition (PVD) coating. The goal of the latter procedure was to provide the surface of the copy with certain optical properties which correspond to the original properties and allow the replicated mechanoscopic marks to become more distinctive.

### 3.1. Mold Making

In a typical RTV process, the mold is cast by encapsulating the master pattern in elastomer (silicone rubber). At this stage, it is important for the mold to be properly designed, allowing for the location of the mold cavity as well as the gating and the flow-off (venting) systems. They are crucial for the proper filling of the mold cavity in the VC process and thus for the production of proper replicas. Their location must also ensure that the molding box is properly filled with elastomer (silicone) during the tool-casting process and prevent the formation of “air traps”—areas in which the remaining air bubbles lead to incorrect reconstruction of the surface of the cast pattern in the mold.

A standard tool-forming RTV process is performed in 4 main steps, as shown in Figure 1—the mold making stage:

1. Preparation of the master pattern, the gating and feeding system, and the molding box;
2. Preparation of the mold material compounds (two compounds of silicone rubber);
3. Casting of the mold (encapsulation of master pattern with silicone rubber);
4. Removal of the master pattern.

**Figure 2:**
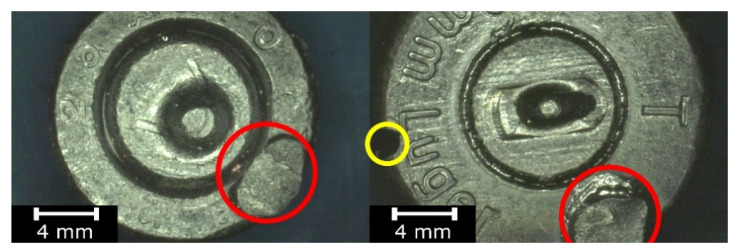
Mark left by the removed gating channel on the surface of the replica (indicated in red) and casting defects (indicated in yellow).

The use of the standard mold-making RTV process causes technological marks to occur on the surfaces of the cast objects, as shown in Figure 2. As is the case for example in die-casting, cast surfaces contain marks due to both material flashes on the parting surfaces of the mold and the design of the gating and feeding system. When replicating such objects as bullets and cartridge cases, additional artifacts on the surface of the copy, introduced as a result of the technological process and visible in Figure 2, prevent the replica from being used in further analyses of mechanoscopic marks and identifications of the related firearms.

**Figure 3:**
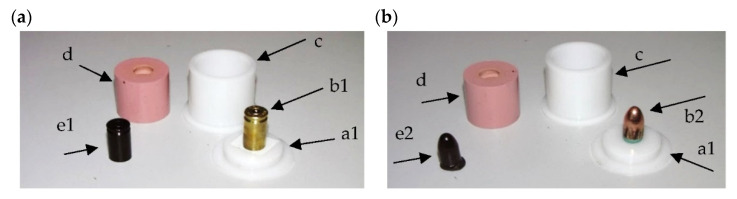
View of the casting molds with cartridge case (**a**) and bullet (**b**) used for testing. Image present the molding box (**c**), parting surface (**a1**), with the position of cartridge case (**b1**), bullet (**b2**), molds (**d**), and cast replicas (**e1**,**e2**).

Due to the above-described limitations of the RTV method in the production of casting molds for the purposes of the replication process, a new approach to the mold preparation process has been proposed. It consists in the use of a simplified single-part tool, without an additional parting surface and the gating and feeding system. Developed for the purposes of replicating the objects in question, the variant of the method also involves the application of a simplified cylindrical molding box adjusted to the size of the replicated object, as illustrated in Figure 3. Due to the elimination of the gating and feeding system, the first step of the developed process only requires the master pattern/replicated object to be secured to the base of the molding box. In the second step of the process, the two components of the silicone rubber (the catalyst/hardener and the base) are mixed. Subsequently, the uncured mold material is degassed in vacuum in order to remove air bubbles formed during the mixing process as a result of high density and viscosity of silicone. During the degasification, a rapid, up to tenfold, increase in the volume of the material is observed. In the third step of the process, after the air bubbles are removed, silicone is poured into the molding box and the mold is cast. The degasification process is then repeated. The curing process is performed in ambient temperature or, depending on the type of silicone, it may be accelerated by placing and holding the uncured mold in a stove. In the fourth step, the master pattern is removed from the ready mold. Unlike in the typical RTV process of mold preparation, in which the removal of the master pattern must be preceded by the cutting of the mold along the parting surfaces provided beforehand, in the newly developed variant this procedure is not necessary, as the master pattern is arranged directly at the front surface of the mold, see Figure 3a.

Figure 3 shows examples of the molding boxes (c), the arrangement of the master patterns (a1) with respect to the parting surface (a), the molds (d), and the cast replicas (e1, e2).

The proposed, simplified system allows the preservation of all of the important marks on the circumferential and frontal surfaces of the master pattern (e.g., the barrel thread—in the case of bullets, or the firing pin or the extractor—in the case of cartridge cases). At the same time it does not require a degassing system, which would leave marks on the cast surfaces. Additionally, it simplifies the replication process by allowing the molds to be filled at atmospheric pressure.

The molds used in this research were made of a two-component silicone Xiameter RTV-4250-S, in accordance with the procedure described above. Physical properties of the mold material are shown in Table 1. The components (both the base and the activator) were combined at a proportion of 10:1 by weight. The mix was degassed in a vacuum chamber at a pressure of 1 × 10^−1^ mbar, and over a time of 90 s. After the already prepared molding box with the master pattern placed in it was filled at atmospheric pressure, the entire part was again placed in a vacuum chamber and degassed using the previous pressure and time settings. After the silicone was cured at a room temperature and over a time of 10 h and after the master pattern was removed, the result was the replication tool ready for use. The RTV tool manufacturing process can be shortened from 10 h to 20 min at a temperature of 50 °C.

The tests were performed on two molds prepared for two objects: the case and the bullet, as shown in Figure 3.

### 3.2. Part Casting

Part casting is the essential part of the replication process, in which the prepared tool is used to produce replicas. During the research works, three variants of this stage were proposed and verified:

1. Vacuum casting (VC),
2. Hybrid atmospheric-vacuum casting (HAVC),
3. Hybrid atmospheric-vacuum-pressured casting (HAVPC).

HAVC and HAVPC are hybrid processes, developed for the replication of objects used in forensic investigations. Hybrid atmospheric-vacuum casting process has three main steps:

1. An atmospheric pressure step, covering both the combination and mixing of the thermosetting polymers, as well as pouring them in the mold;
2. A vacuum step, during which the thermosetting polymer poured into the mold is briefly degassed;
3. A polymerization step, performed at atmospheric pressure and at ambient temperature or at an elevated temperature in order to shorten the curing time.

Unlike in the VC process, in the HAVC process both the combining and the mixing of the polymer components used for the production of the replica, as well as the filling of the mold, are performed at atmospheric pressure. As a result, the molds can have a less complicated design without gating and venting systems, as illustrated in Figure 3 and Figure 7. In order to remove the air bubbles and pores from the cast replicas, after the mold is filled with the polymer in the second HAVC step, it is vacuum-treated, as in the case of a standard VC variant. The third step of the process, polymerization, is also performed in atmospheric pressure, as in the VC process. This step may be performed at ambient temperature or in elevated temperature in order to shorten the curing time.

The third of the analyzed variants—HAVPC is performed in four steps:

1. An atmospheric step, covering both the combination and mixing of the thermosetting polymers, as well as pouring them in the mold;
2. A vacuum step, covering preliminary degasification by removing/closing the pores and their transfer to the near-surface area;
3. A pressure step, provided in order to reduce and remove all of the near-surface pores;
4. A polymerization step performed at atmospheric pressure and, as in HVAC, at an ambient or elevated temperature.

This method was developed as an evolution of the HVPC variant, in order to eliminate its low efficiency of the single vacuum degasification stage, which is a flaw resulting from the tendency of polyurethane resins for foaming in vacuum treatment causing pores to form in the near-surface area (the contact area between the mold and the cast part). This phenomenon may be due to an incorrect selection of vacuum casting parameters (e.g., component mixing or degasification durations) or an increased ambient humidity in the location where the process is performed. The additional pressure treatment in HAVPC was aimed at reducing the quantity and size of the pores formed as a result of the second (vacuum) stage of the process.

**Figure 4:**
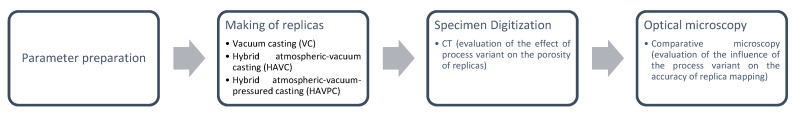
Schematic diagram of the tests in stage 2.

The tests being part of the second stage of the replication process (part casting) were performed in four steps, as illustrated in Figure 4.

The parts were cast with the use of RenLam M1 resin and Ren HY 956 activator. The details are shown in Table 2. In order to facilitate the use of optical microscopy in the analysis of the cast specimens, a black coloring agent was added to the resin, in a proportion by weight of 1:20, respectively. The selection of the coloring agent depends on the expected result and on own requirements. The palette of colors available for use with epoxy resins and having the greatest visible light absorbance covers brown colors (RAL 8016) or black colors (RAL 8022). For this reason, they are preferred in the case when the material is sent for tests as part of cooperation between different laboratories. Both colors were accepted as best during the microscopic examinations.

13 series of specimens were made for the three described methods (VC, HAVC, HAVPC) using the parameter sets described in Table 3. As part of each series, two sets of both cartridge cases and bullets were cast. The variable casting parameters included: feeding pressure, degasification pressure and time, pressure treatment time and pressure, as well as mold temperature. All of the parts were cast in identically prepared molds, in an open system, as described in Section 3.1.

### 3.3. Post-Processing

The last stage of the replication process consists in the application of a PVD coating. The advantages of the method include good cohesion of the PVD layer, which does not peel off, high durability and low process temperature, which does not lead to thermal deformations. As a variant of the PVD method, sputtering was performed in vacuum, and the coating material used was Au. This method is advantageous in that it ensures high cohesion of the applied coatings to the substrate, a uniform coating and low process temperature. The parameters of the process are shown in Table 4. The coatings were applied with the use of a Sputter Coater 7620 vacuum machine.

The quality of the prepared replicas was evaluated with the use of an industrial computed tomography system Zeiss Metrotom 1500. The manufacturing accuracy of the replica specimens was evaluated with the use of a light microscope Keyence VHX-600 and of the Automated Ballistic Identification System ARSENAŁ used by the Polish police.

The research set-up for manufacturing the replicas of cartridge cases and bullets comprised:

- A KLM V400 A vacuum chamber for producing the molds and replicating the master patterns,
- A POL-EKO SLM 15 STD stove for preparing the molds and the cast parts,
- A pressure chamber for further treatment of the cast parts,
- A Sputter Coater 7620 machine for sputtering the PVD coatings.

## 4. Results and Discussion

### 4.1. Assessment by Industrial Computed Tomography (CT)

**Figure 5:**
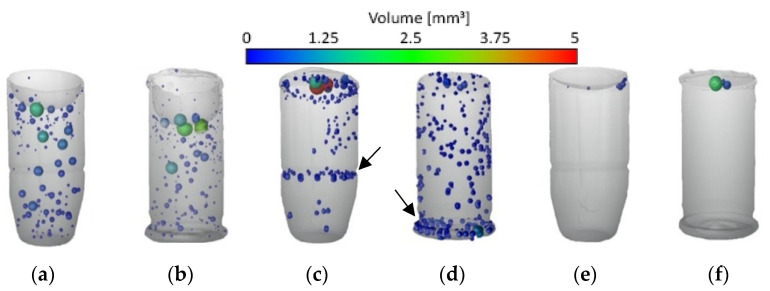
3D models of specimens reconstructed with computed tomography Images of series 2 (VC) (**a**,**b**); series 7 (HAVC) (**c**,**d**), series 13 (HAVPC) (**e**,**f**).

The specimens (replicas of cartridge cases and bullets manufactured in the three analyzed process variants) were subjected to a computer tomography examination in order to verify the size and the character of their porosity. Figure 5 shows the qualitative results of CT examinations in the form of views (images) for the reconstructed 3D models of specimens from series 2, 7, and 13. They are representative of the remaining CT examination results for specimens from series produced with the use of respective process variants: images of specimens from series 2 (VC) are similar to images of specimens from series 1, 3, 4, and 5, images of specimens from series 7 (HAVC) are similar to images of specimens from series 6, 8, 9, and images of specimens from series 13 (HAVPC) are similar to images of specimens from series 10–12.

The specimens of series 1–5, produced in the VC process, showed pores uniformly distributed in the volume of the specimen, for the replicas of both the cases and the bullets, as shown in Figure 5a,b. The specimens of series 6–9, produced in the HAVC process, showed a tendency for the pores to gather in near-surface layers and along the circumferences indicated with arrows in Figure 5c,d. In the case of the specimens of series 13, shown in Figure 5e,f, a significant reduction in the number of pores was observed. They were found only in the upper parts of the specimens.

Moreover, the CT examinations allowed the identification of both the number and the shape of the pores, as presented in Table 5. The porosity was calculated as a relationship between the volume of the reconstructed object without voids and the total volume of the reconstructed object. The quantitative results of specimen examinations of pores size, their distribution and sphericity, were also similar in the series of specimens produced with the same process variant, and therefore Table 5 contains the results for specimens from series 2, 7 and 13, which are representative of the analysis results for the remaining specimens produced with the same process. For the VC process the difference between the highest and lowest measured porosity was up to 10%, for the HAVC process it was 14%, and for the HAVPC process 8%.

The shape of the pores was analyzed with the use of a sphericity coefficient *S*, which is *S* = 1 for a sphere:

(1) $$S=\frac{\pi^{1/3}{\left(6V\right)}^{2/3}}{A}$$

where *V* is the defect volume and *A* is the defect surface.

**Figure 6:**
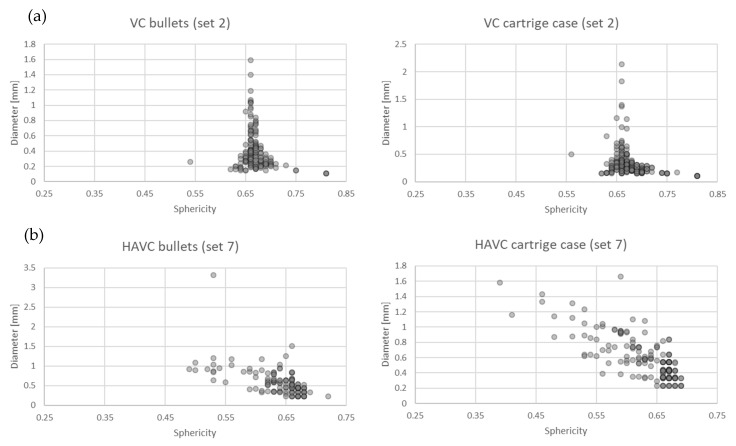
Shape and dimensions of the pores in specimens from series (**a**) 2 (VC) and (**b**) 7 (HAVC).

Figure 6 shows the distribution of the sizes and shapes of the pores for series 2 (VC) and 7 (HAVC). Due to their neglectable porosity, specimens from series 13 have not been included in this analysis.

The identified porosity of specimens from series 2 and 7 was similar (Table 5). Represented as series 2, the specimens produced with the VC process contained pores similar with respect to the sphericity coefficient S, which was approximately 0.65. The pore size was within the range of 0.1–1.6 mm, with the majority not exceeding 0.8 mm, as illustrated in Figure 6a. In the case of series 7, which represents specimens produced with the HAVC method, more significant shape differences can be observed, with the S coefficient being 0.4–0.7. As shown in Figure 6b, the pores in the specimens from this series had a similar size range of 0.2–1.5 mm, with the majority not exceeding 1 mm. The average pore size determined for the specimens from series 2 was 0.37 mm, and for the specimens from series 7 was 0.58 mm.

CT analyses were also performed for molds used in the research. In this case, pores were observed neither in the material of the molds nor on their surfaces. The CT results confirmed that the manufacturing process was correct.

### 4.2. Analysis of the Accuracy of Geometric Reproduction

In the subsequent stage of the analyses, a macroscopic examination of the specimens was performed with the use of a light microscope. As in the case of the CT examinations, the analysis was performed for specimens representing three variants of the replication process, i.e., series 2, 7, and 13. The results are shown in Table 6.

**Figure 7:**
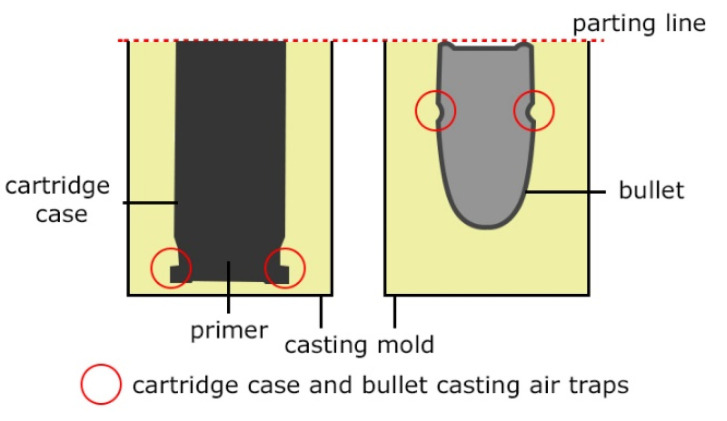
Schematic view of areas in the molds responsible for air traps and surface defects of the bullets and the cartridge case replicas.

As demonstrated in the CT analyses, replication with the VC process resulted in the formation of air bubbles on the surfaces of the specimens. In the specimens from series 2, and in the remaining series 1–9, they were located in areas indicated with arrows in the images of Table 6. In the case of the cartridge cases, the defects were observed in the vicinity of the extractor groove, and in the case of the bullets—in the narrowing on the circumferential side surface. Similar defects can be observed in the case of the specimens from series 7, made with the HAVC process. In this case, the number of pores on the surface is smaller. However, they have a greater volume, as visible in Figure 5 and Figure 6. The presence of pores on the surfaces of the specimens from series 2 and 7 may be related to the methods used in casting the replicas. The mold design, schematically shown in Figure 7, results in the formation of negative inclinations on the cylindrical forming surfaces of the molds, indicated with circles in the areas of undercuts on the reconstructed objects. The resulting so-called air traps prevented the air bubbles formed in the vacuum from being transported towards the surface of the mold.

The broadening of the HAVC process with an additional, high-pressure stage (HAVPC) caused the pores on the external surfaces of the cast parts to be completely reduced in both the number and the size, as illustrated in Figure 5e,f. The residual porosity observed in the upper part of the cast objects is not important from the perspective of replicating mechanoscopic forensic marks on cartridge cases and bullets. The analyses also involved verification of the possibility to skip the vacuum degasification stage in the replication process. However, in all of the tests, this simplification resulted in the formation of defects (pores) concentrated in locations (air traps) shown in Figure 7. The degasification stage facilitates the removal of air bubbles from the cast objects, their transportation to the upper part of the cast objects, and their concentration in the near-surface area. It also facilitates the removal of pores in the subsequent stage of pressure treatment. In the HAVC and HAVPC variants, unlike in the VC process, the vacuum had a lower value of 250–500 mbar in order to prevent excessive increase in the volume of the polymer cast in the mold and consequently its overspill from the mold cavity.

As shown in Figure 5 and in Table 6, the specimens from series 13, produced with the use of the HAVPC process, had no pores on the external surfaces of the replicated objects and had only residual internal porosity. Previous studies [5,8,9,10] of the VC process were mainly aimed at determining the accuracy of the geometric representation and confirming the method’s applicability in replicating selected geometric structures. The results of the work presented in the article using five different sets of VC process parameters did not confirm the conclusions of D.Y. Zhao et al. on the crucial role of vacuum parameters in avoiding defects on the surfaces. The applied variants of VC and HVAC did not allow to achieve casts without surface defects. The porosity of the replicas made for both variants was similar, as presented in Table 5. Due to the longer vacuum exposure in the VC variant, pores were formed in the cast material’s whole volume. Shortening the time of vacuum action in the HAVC variant to the degassing stage reduced the blisters’ size, formed mainly on the surfaces of the specimens. They were also smaller than the pores formed in the VC process. Complete elimination of the vacuum degassing stage is not possible in the analyzed processes due to the necessity to remove bubbles formed as a result of mixing the components of the cast resin, Kuo C-C [2], and to obtain high accuracy of replication. An additional treatment step of elevated pressure in the HAVPC variant allowed closing the small pores formed in the HAVC variant.

**Figure 8:**
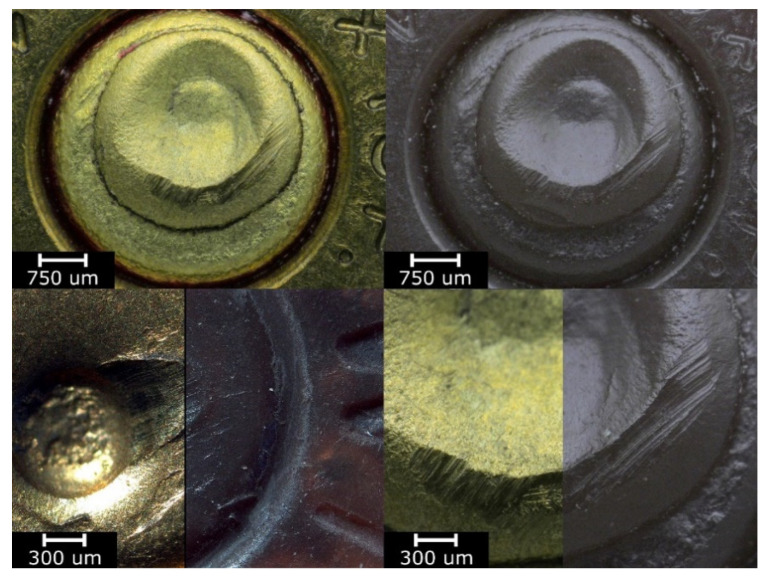
View of the specimens from series 13 (replicas) compared to the original objects. Images of the original objects are on the left, the replicas are on the right. Images were recorded with a comparison microscope.

Examinations of the specimens in this series performed under a comparison microscope demonstrated that the process allowed an accurate reconstruction of all of the characteristic (mechanoscopic) marks present on the surface of the original object. This is illustrated in Figure 8. The images show optical differences between the surfaces of the original and reconstructed objects.

**Figure 9:**
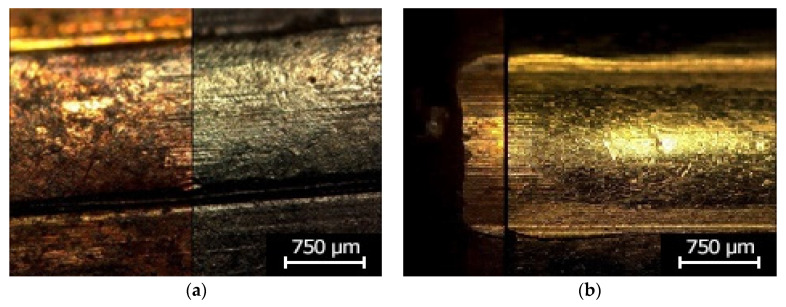
Images of the characteristic properties of the surface marks: (**a**) bullet and the replica coated with Au—10 nm, (**b**) bullet and the replica coated with Au—10 nm.

Following the research plan, the specimens properly produced in the HAVPC process were subjected to post-processing by coating them with an additional PVD layer, in accordance with the parameters shown in Table 4. The objective was to provide them with optical properties similar to those of the original objects and as a result to facilitate their analysis under a comparison microscope during the identification process. The specimens with the applied PVD coatings were analyzed with the use of a comparison microscope of the Automated Ballistic Identification System used in Polish forensic laboratories. Sample images from this analysis are shown in Figure 9. The comparison of the original bullets and cases with their copies demonstrates that all of the characteristic micro and macro marks present on the surfaces of the original objects were reconstructed in their replicas. Moreover, the surfaces of the replicas preserved the optical properties of the original objects, and as a result all of the characteristic marks present on the original objects are also visible on their replicas, which in fact facilitated the identification process.

The analysis results indicate that an additional PVD coating improved the optical characteristics of the replica surfaces, their reflectability, and original color mapping. These properties are particularly important when using a light/comparison microscope in forensic examinations. The microscopic analysis demonstrated that the cast cartridge cases and bullets have accurately reconstructed marks of the firing pin, the foregrip, the ejector, and the barrel thread, as well as the characteristic points such as scratches or indentations. This is shown in Figure 8 and Figure 9. These characteristic marks enable examinations of replicated cartridge cases and bullets in order to perform individual identifications of firearms.

## 5. Conclusions

The aim of the analyses presented in this article was to develop a complementary forensic mark replication process which would require short preparation time, prove simple to perform, and yield highly repeatable results.

The proposed new approach to using the RTV process in the production of simplified casting molds free from the gating, feeding, and venting systems allowed the preparation of replicas without additional artifacts introduced on the mold surface. The developed approach allowed not only the tool preparation to be simplified and shortened but also the entire process time to be shortened from 10 h to 1.5 h.

The research works covered tests of the VC-based replica manufacturing method as well as its proposed hybrid variants: HAVC and HAVPC. The implementation of a hybrid process with additional stages consisting in filling the mold at atmospheric pressure allowed a simplified mold design, which was developed in the first stage of the research. On the other hand, the pressure treatment stage allowed the number and the size of the pores on the cast surfaces to be reduced. The final variant of the process allowed the tendency of polyurethane resins for foaming in vacuum treatment, which causes bubbles in the near-surface area of the cast parts, to be minimized.

Examinations of the replica specimens, performed with the use of computed tomography and light microscopy, confirmed that the stages of the HAVPC process were realized correctly.

The sputtering of an additional PVD coating significantly increased the optical properties of the replicas, enabling them to reach the level of the original objects.

The developed replication process allowed the replicas to be prepared without additional defects or artifacts introduced on the surfaces of the replicas. Comparative images of the surfaces of both the original and replicated objects demonstrated that the individual properties (the micro and macro mechanoscopic marks present on the surfaces of the cartridge cases and bullets) were reconstructed accurately. The method here presented allows the preparation of copies, their analysis, and the identification of similar objects in the systems used by forensic laboratories, e.g., in the Automated Ballistic Identification System.

## Figures and Tables

**Table 1:** Physical properties of the mold material (silicone type Xiameter RTV-4250-S).

	Silicone	Catalyst	Mix
Mixing proportion (by weight)	10	1	
Low Temperature Brookfield Viscosity (mPas)	26,000	140	13,000
Density at 25 °C (g/cm^3^)	-	-	1.12
Working life at 23 °C (for 150 g)	-	-	105 min
Mechanical properties at 23 °C (1)			
Hardness after 24 h	-	Shore A	61
Elongation at break	-	%	850
Breaking strength	-	MPa	7
Tearing strength	-	kN/m	23

**Table 2:** Physical properties of the resins used for part casting (RenLam M1 and Ren HY 956).

	RenLam M-1	Ren HY 956 (Activator)	Mix
Mixing proportion (by weight)	5	1	
Form	Liquid	Liquid	Liquid
Working life at 25 °C (for 500 g)			30 min
Viscosity at 25 °C mPas	1250–1600	370–470	1200
Density g/cm^3^	1.1	1.0	1.1

**Table 3:** Parameters of the series of specimens manufactured by Vacuum Casting (VC), Hybrid atmospheric-vacuum casting (HAVC), and Hybrid atmospheric-vacuum-pressured casting (HAVPC) processes.

	Series No.	Vacuum Casting			Pressure Casting		Mold Preparation Temperature (°C)
		Feeding Pressure (mbar)	Degasification Pressure (mbar)	Degasification Time (s)	Pressure (bar)	Time (min)	
VC	1	1 × 10^−1^	atm	90	-	-	20
	2	1 × 10^−1^	1 × 10^−1^	90	-	-	20
	3	1 × 10^−1^	1 × 10^−1^	90	-	-	60
	4	1 × 10^−1^	75	90	-	-	20
	5	1 × 10^−1^	75	90	-	-	60
HAVC	6	atm	250	90	-	-	60
	7	atm	500	90	-	-	60
	8	atm	400	120	-	-	60
	9	atm	500	120	-	-	60
HAVPC	10	atm	400	120	1	165	60
	11	atm	400	120	2	90	60
	12	atm	400	120	2	60	60
	13	atm	500	120	2	60	60

**Table 4:** Parameters of the Physical Vapor Deposition (PVD) coating process.

Application of PVD Coatings	
Current intensity	18 mA
Coating speed	7.5 nm/1 min
Coating thickness	10 µm
Coating (target) material	Au

**Table 5:** Results of porosity examinations for the cast specimens from series 2 (VC), 7 (HAVC), and 13 (HAVPC).

Series No.	$\bar{X}\;\mathrm{Porosity}\;(\%)$	Standard Deviation	Confidence Interval	Location of the Pores	
				Internal	On the Surface Layer
2	1.360	0.033	0.034	+	+
7	1.293	0.086	0.089	+	+
13	0.097	0.097	0.054	+	-

Confidence interval for population mean Student’s T distribution a-0.05.

**Table 6:** Macroscopic images of external surfaces for selected representative series of case and bullet replicas. The arrows in the images indicate defects (pores) on the specimen surfaces. In the case of series 13, the images also show specimens with PVD coatings.

Series	Bullets	Cartridge Case
2	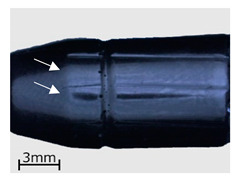	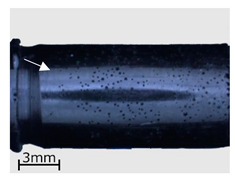
7	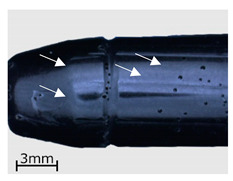	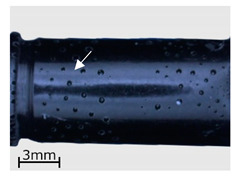
13	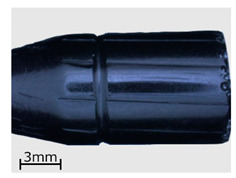	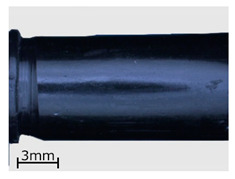
	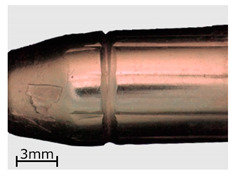	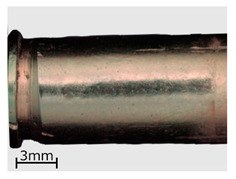

## Data Availability

The data presented in this study are available on request from the corresponding author.
